# The Effect of Exercise Intervention on Cognitive Function and Quality of Life With Autism Spectrum Disorder: A Systematic Review and Meta-Analysis

**DOI:** 10.62641/aep.v53i4.2040

**Published:** 2025-08-05

**Authors:** Benben Cai, Yingying Miao, Jiajia Zhao, Xiaoming Ying, Weiqiang Lin

**Affiliations:** ^1^Department of Pediatrics, Taizhou First People's Hospital (Huangyan Hospital, Wenzhou Medical University), 318020 Taizhou, Zhejiang, China

**Keywords:** physical exercise, children with autism spectrum disorder (ASD), social communication, social cognition, sleep, behavioral aspects, motor skills, quality of life

## Abstract

**Background:**

Physical exercise may confer benefits on cognitive function and quality of life in children with autism spectrum disorder (ASD). However, the evidence has not been co mprehensively synthesized. This study aimed to investigate the effect of exercise intervention on cognitive function and quality of life with ASD, and provide evidence to support the scientific use of exercise interventions in practice.

**Methods:**

We systematically searched major databases from inception to November 2023 for randomized trials and observational studies examining exercise interventions in children with ASD. Mean differences (MDs) with 95% confidence intervals (CIs) were calculated using random-effects models. Heterogeneity was assessed using the I^2^ statistic. Risk of bias was evaluated with the Cochrane tool.

**Results:**

Fourteenth studies were included. Meta-analysis of 8 randomized trials found a small but significant effect of exercise on social communication (MD: 1.42, 95% CI: 0.21 to 2.6322, *p* = 0.02, I^2^ = 29%). The effect on social cognition was also significant (MD: 1.99, 95% CI: 0.18 to 3.80, *p* = 0.03, I^2^ = 0%). Influential analysis identified 2 studies as outliers. Leave-one-out analysis showed meta-analysis conclusions were robust. The included studies consistently demonstrated benefits of exercise on sleep, behavioral aspects, motor skills, quality of life, and other outcomes.

**Conclusions:**

This meta-analysis provides evidence that exercise interventions may improve core symptoms and functional outcomes in children with ASD. However, small sample sizes and heterogeneity indicate cautious interpretation. Further adequately powered trials are needed to establish optimal exercise programs for managing ASD.

## Introduction

Autism spectrum disorder (ASD) is a neurodevelopmental condition characterized by persistent deficits in social communication and social interaction, along with restricted and repetitive patterns of behavior. The worldwide prevalence of ASD has increased substantially over the past few decades, affecting around 1 in 160 children globally [1]. In addition to the core symptoms, children with ASD frequently have comorbid conditions like intellectual disability, language impairment, and psychiatric disorders, which can further exacerbate the functional deficits. Consequently, ASD poses significant challenges for the affected individuals, families, and society.

In recent years, there has been a growing interest in exploring complementary and alternative interventions to alleviate some of the impairments associated with ASD. Physical exercise has emerged as a promising approach, with studies demonstrating its benefits on motor skills, cognitive function, and behavioral aspects in children with ASD. Besides cognitive function, quality of life is another major area of deficit in children with ASD [2]. Along with the core symptoms, children with ASD struggle with physical health issues, sleep problems, anxiety, depression, attention deficits, and other challenges that can substantially lower their quality of life [3]. However, regular physical activity has been associated with better subjective well-being and life satisfaction in both typically developing populations [4] and developmental disorders like ASD [5]. Exercise interventions may not only improve physical fitness but also enhance psychological aspects like self-esteem, motivation, and mood in children with ASD.

While individual studies have indicated beneficial effects, there has been no comprehensive synthesis of the current evidence. A systematic review and meta-analysis can provide a rigorous summary of the existing research on this topic. By statistically combining the results from multiple studies, a meta-analysis offers enhanced precision and power for detecting genuine effects of exercise interventions on cognitive function and quality of life outcomes in children with ASD [6]. Therefore, the study aimed to comprehensively review and analyze existing randomized controlled trials to explore the potential impact of exercise interventions on enhancing cognitive function and improving quality of life in children diagnosed with ASD. By synthesizing the research findings, we intended to provide a solid theoretical basis for designing tailored exercise prescriptions to effectively address these critical issues in the pediatric ASD population.

## Materials and Methods

### Literature Search Strategy

We systematically searched the PubMed, Embase, Web of Science, Cochrane Library, PsycINFO, CNKI, Wanfang and CQVIP databases from inception to November 2023 to identify relevant studies on the effect of exercise interventions on outcomes in children with ASD. The search terms included controlled terms (MeSH and Emtree) as well as free text words for concepts related to ASD (e.g., autism spectrum disorder, ASD, autism, Asperger’s), exercise (e.g., exercise, physical activity, aerobic, training), and study design (e.g., clinical trial, controlled study, randomized), see Table 1 for detailed search strategy. Search strategies were tailored to each database. Reference lists of included studies and relevant reviews were hand-searched for additional eligible studies. We applied no language or date restrictions.

**Table 1.** **Search strategy**.

1. Search strategy for PubMed		
	#1	((((((((((autism spectrum disorder[MeSH Terms]) OR (autism spectrum disorder[Title/Abstract])) OR (ASD[MeSH Terms])) OR (autism[Title/Abstract])) OR (autism[MeSH Terms])) OR (Asperger’s[MeSH Terms])) OR (Asperger’s[Title/Abstract])) OR (autism[Title/Abstract])) AND ((((((((exercise[MeSH Terms]) OR (exercise[Title/Abstract])) OR (physical activity[MeSH Terms])) OR (physical activity[Title/Abstract])) OR (aerobic[MeSH Terms])) OR (aerobic[Title/Abstract])) AND ((((((((clinical trial[MeSH Terms]) OR (clinical trial[Title/Abstract])) OR (controlled study[MeSH Terms])) OR (controlled study[Title/Abstract])) OR (randomized[MeSH Terms])) OR (randomized[Title/Abstract])) OR (RCT[MeSH Terms])) OR (RCT[Title/Abstract]))
2. Search strategy for Web of Science		
	#1	Topic Search
	#2	((((TS=(exercise)) OR TS=(physical activity)) OR TS=(aerobic))
	#3	((((TS=(clinical trial)) OR TS=(controlled study)) OR TS=(randomized))
	#4	#1 AND #2 AND #3
3. Search strategy for Cochrane Library		
	#1	MeSH descriptor: [autism spectrum disorder] explode all trees
	#2	MeSH descriptor: [exercise] explode all trees
	#3	(autism spectrum disorder or ASD or autism or Asperger’s):ti,ab,kw
	#4	(exercise or physical activity or aerobic):ti,ab,kw
	#5	(clinical trial or controlled study or randomized):ti,ab,kw
	#6	#1 or #3
	#7	#2 or #4
	#8	#5 and #6 and #7
4. Search strategy for PsycINFO		
	#1	SU autism spectrum disorder OR SU ASD OR SU autism OR SU Asperger’s
	#2	DE exercise OR DE physical activity OR DE aerobic
	#3	SU clinical trial OR SU controlled study OR SU randomized
	#4	#1 AND #2 AND #3
5. Search strategy for Embase		
	#1	(‘autism spectrum disorder’:ti,ab,kw OR ‘ASD’:ti,ab,kw OR ‘autism’:ti,ab,kw OR ‘Asperger’s’:ti,ab,kw) AND(‘exercise’:ti,ab,kw OR ‘physical activity’:ti,ab,kw OR ‘aerobic’:ti,ab,kw) AND (‘clinical trial’:ti,ab,kw OR ‘controlled study’:ti,ab,kw OR ‘randomized’:ti,ab,kw OR ‘RCT’:ti,ab,kw)

ASD, autism spectrum disorder; RCT, randomized controlled trial.

### Eligibility Criteria

We included randomized controlled trials (RCTs) and controlled clinical trials (CCTs) examining the effects of exercise or physical activity interventions on cognitive, behavioral, psychosocial or physiological outcomes in children diagnosed with ASD. The diagnosis of ASD was defined according to the Diagnostic and Statistical Manual of Mental Disorders-5 (DSM-5) [7], the Autism Diagnostic Interview-Revised (ADI-R) or other standardised diagnostic criteria. Studies of participants with other types of disabilities were excluded if they could not distinguish between specific data on people with ASD. The exercise interventions included structured or unstructured workouts, physical activities, or exercise regimens. The comparison group could receive no treatment, standard care, or an alternative therapy. Studies had to report quantitative outcome data that could be extracted for meta-analysis. We excluded studies with inadequate control groups, insufficient data, overlapping populations, and those measuring acute post-exercise effects. Studies that did not involve any comparison group or did not report any comparison results between groups were excluded from the meta-analysis.

### Study Selection and Data Extraction

Two reviewers independently screened the titles, abstracts and full texts of retrieved studies against the eligibility criteria. Disagreements were resolved by consensus or consultation with a third reviewer. A standardized form was used to extract data on study characteristics (design, country, sample size), participant details (age, diagnosis), intervention details (type, frequency, duration), and outcome data including means, standard deviations, and sample sizes for intervention and control groups. Corresponding authors were contacted for missing information.

### Risk of Bias Assessment

Two reviewers independently assessed the methodological quality and risk of bias in included studies using the Cochrane Collaboration Risk of Bias tool. This covers the adequacy of randomization, allocation concealment, blinding, completeness of outcome data, selective reporting, and other sources of bias. Each domain was judged as low, unclear or high risk of bias. Discrepancies were resolved through discussion.

### Statistical Analysis

Meta-analyses were performed using the meta and dmetar packages in R software 3.3.3 (R Foundation for Statistical Computing, Vienna, Austria) [8]. Mean differences (MDs) with 95% confidence intervals (CIs) were calculated as the effect sizes for continuous outcomes using inverse-variance random-effects models. Heterogeneity was assessed using the I^2^ statistic. I^2^ < 25% was considered low heterogeneity, 25% ≤ I^2^ < 50% was considered moderate heterogeneity, and I^2^ ≥ 50% was considered high heterogeneity. Publication bias was evaluated through funnel plots and Egger’s regression asymmetry test [9]. Influential analysis was conducted using the Baujat plot [10] and Galbraith plot [11]. Leave-one-out analysis assessed the influence of individual studies. The Graphic display of study heterogeneity (GOSH) diagnostics explored clustering and outliers using methods like DBSCAN and Gaussian mixture models. This study was reported following the PRISMA guidelines (**Supplementary file 1**).

## Results

### Characteristics of Included Studies

**Fig. 1.**
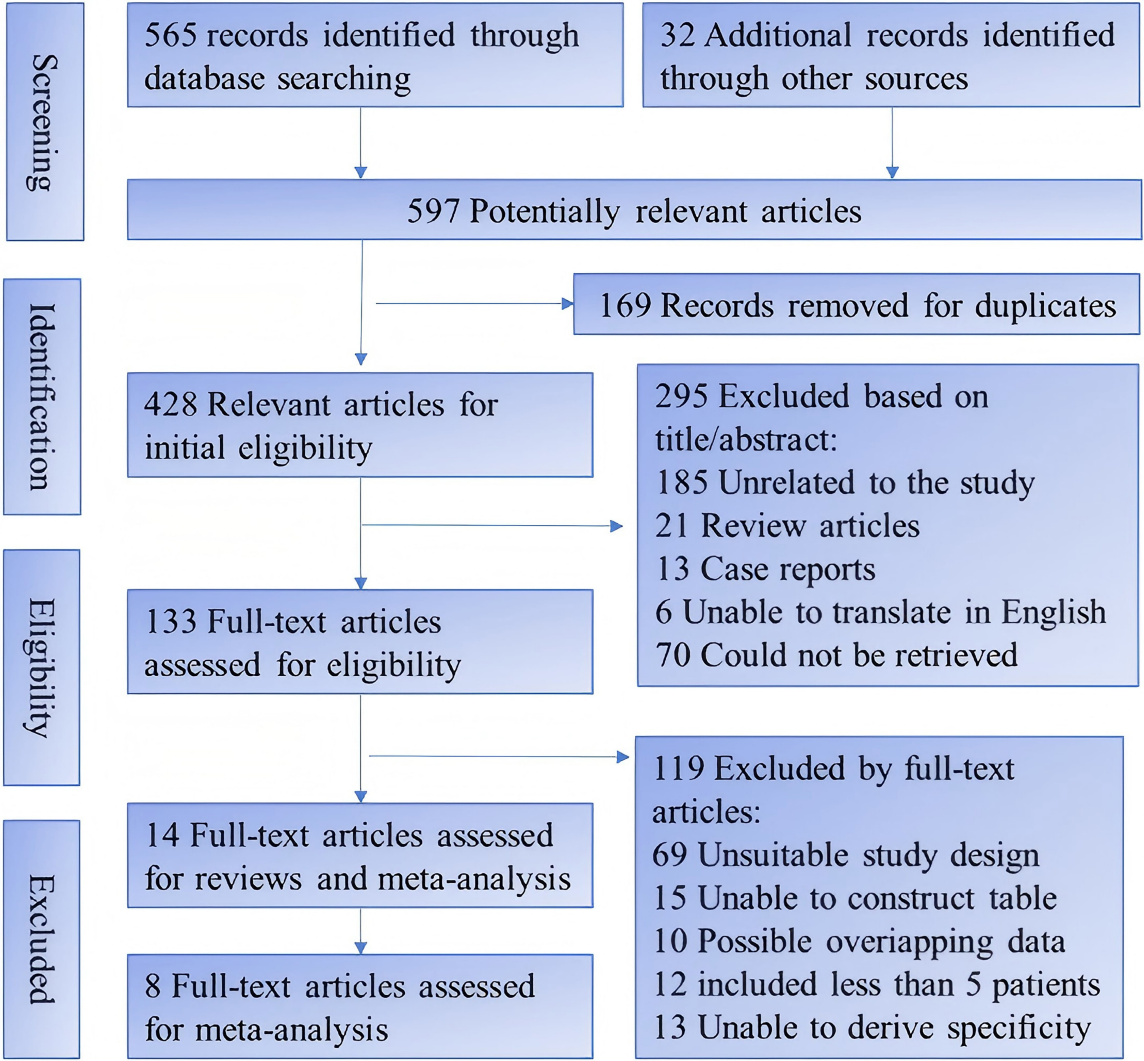
**Flow chart of study selection**. Note: Other sources refer to reference lists of included studies and relevant reviews.

The study selection process followed the PRISMA guidelines, which are a set of standards for reporting systematic reviews and meta-analyses. The initial search of the databases and other sources yielded 597 potentially relevant articles, which were screened for duplicates and eligibility based on their titles and abstracts. After removing 169 duplicates and 295 irrelevant or unavailable articles, 133 full-text articles were assessed for eligibility based on the inclusion and exclusion criteria. Out of these, 119 articles were excluded for various reasons, such as unsuitable study design, insufficient data, or overlapping data. The remaining 14 articles were assessed for quality and risk of bias, and 8 articles were included in the meta-analysis. The flow diagram of the study selection process is presented in Fig. 1.

The findings across these studies consistently demonstrated the therapeutic benefits of physical activities in improving various aspects of life for individuals with ASD. In Switzerland, a study involving aerobic exercise training (AET) and motor skill training (MST) showed notable improvements in sleep, motor skills, and mood among children with ASD. Similarly, in China, a 12-week mini-basketball training program (MBTP) significantly enhanced physical fitness and social communication skills in preschool children with ASD. This was further corroborated by another Chinese study where the MBTP also improved executive functions and core symptoms such as social communication impairment and repetitive behavior in preschoolers with ASD. In Hong Kong, China, the practice of Chinese Chan-based mind-body exercise, Nei Yang Gong, led to greater improvements in self-control, reduced severity of autistic symptoms, and enhanced behavioral control compared to Progressive Muscle Relaxation (PMR) technique. Additionally, in Iran, combined physical training resulted in significant social skill enhancements, including reduced stereotypic behavior and improved communication, as well as better physical fitness. Karate techniques training in another Iranian study showed a marked reduction in communication deficits in children with ASD. In Italy, an innovative swimming program positively impacted interpersonal skills, with sustained improvements seen at follow-up. This program also led to gains in autonomy and reductions in negative behaviors. A quasi-experimental trial from China reported that the MBTP significantly enhanced social communication in preschool children with ASD, as well as increased functional connectivity within brain regions of the executive control network (ECN). Dance Movement Psychotherapy (DMP) in the United Kingdom yielded substantial improvements in social and emotional well-being, evidenced by reduced Social Communication Questionnaire (SCQ) scores. An exercise program from China, which included aerobic, resistive, and neuromuscular exercises, led to significant reductions in fat mass and improvements in Autism Treatment Evaluation Checklist (ATEC) scores. In Germany, dance movement therapy incorporating mirroring improved well-being, body awareness, self-other differentiation, and social skills in young adults with ASD. From the USA, a cross-sectional study found that various physical activities like yoga and golf promoted health-related quality of life in young adults with and without ASD. Lastly, a Portuguese study indicated that a 48-week exercise-based intervention improved the metabolic profile, autism traits, and parent-perceived quality of life in children with ASD. Overall, these studies collectively highlight the significant impact of diverse physical activities and interventions on enhancing the quality of life, social skills, physical fitness, and behavioral aspects in individuals with ASD, suggesting the immense potential of these interventions in ASD therapy (Table 2, Ref. [12, 13, 14, 15, 16, 17, 18, 19, 20, 21, 22, 23, 24, 25]).

**Table 2.** **Summary of studies included in the meta-analysis**.

Study	Country/Region	Study Design	Sample size		Mean age/years		Intervention type	Main findings
			Experimental group	Control group	Experimental group	Control group		
Brand *et al*., 2015 [12]	Switzerland	Experimental research	6	N/A	10 ± 2.34	N/A	Aerobic exercise training (AET) and motor skill training (MST)	The study found that in children with ASD, a combination of aerobic exercise training (AET) and motor skill training (MST) can significantly improve sleep, motor skills, and mood, suggesting the potential therapeutic value of this intervention for children with ASD.
Cai *et al*., 2020 [13]	China	RCT	30	29	5.03 ± 0.64	4.56 ± 0.84	Mini-basketball training program	The study found that a 12-week mini-basketball training program (MBTP) significantly enhanced physical fitness, including speed-agility and muscular strength, and fostered improved social communication skills in preschool children with ASD, highlighting the potential therapeutic benefits of physical exercise interventions for this population.
Chan *et al*., 2013 [14]	Hong Kong, China	RCT	20	20	11.28 ± 3.90	12.42 ± 3.25	Mind-body exercise	The study found that in children with ASD, the practice of Chinese Chan-based mind-body exercise, Nei Yang Gong, yielded significantly greater improvements in self-control, reduced the severity of autistic symptoms, and enhanced control over temper and behaviors compared to the conventional Progressive Muscle Relaxation (PMR) technique.
Haghighi *et al*., 2023 [15]	Iran	RCT	8	8	9.00 ± 1.31	8.13 ± 1.36	Combined physical training	The study found that combined physical training (CPT) resulted in significant improvements in social skills, manifested by reduced stereotypic behavior and enhanced communication, as well as enhanced physical fitness, encompassing increased handgrip strength, upper and lower body power, flexibility, balance, and agility in children with ASD.
Bahrami *et al*., 2016 [16]	Iran	Prospective cohort study	15	15	9.20 ± 3.32	9.06 ± 3.33	Karate techniques training	The study found that karate techniques training for children with ASD resulted in a marked and sustained reduction in communication deficits.
Wang *et al*., 2020 [17]	China	RCT	18	15	5.11 ± 0.65	4.70 ± 0.70	Mini-basketball training program	The study found that 12-week mini-basketball training program (MBTP) yielded substantial improvements in executive functions and core symptoms, encompassing social communication impairment and repetitive behavior, among preschoolers with ASD.
Zanobini and Solari, 2019 [18]	Italy	RCT	13	12	5.69 ± 1.27	5.42 ± 1.54	Swimming program	The study found that an innovative swimming program demonstrated a positive impact on interpersonal skills in children with ASD. Notably, improvements persisted at follow-up, extending beyond interpersonal skills to encompass gains in autonomy and reductions in negative behaviors.
Yang *et al*., 2021 [19]	China	Quasi-experimental trial	15	15	4.67 ± 0.70	5.03 ± 0.55	Mini-basketball training program	The study found that 12-week mini-basketball training program (MBTP) significantly enhanced social communication (SC) in preschool children with ASD, as reflected in improved SRS-2 scores. Moreover, the MBTP fostered heightened functional connectivity within specific brain regions of the executive control network (ECN).
Aithal *et al*., 2021 [20]	United Kingdom	Crossover design study	26	N/A	10.65	N/A	Dance Movement Psychotherapy	The study found that Dance Movement Psychotherapy (DMP) intervention for children with ASD yielded substantial improvements in social and emotional well-being, as evidenced by reduced Social Communication Questionnaire (SCQ) scores.
Ye *et al*., 2019 [21]	China	Prospective study	24	N/A	11–14 years	N/A	8-week exercise program that included aerobic, resistive, and neuromuscular exercises.	The study found that the exercise program resulted in significant reductions in fat mass and improvements in ATEC scores, suggesting that exercise-based intervention may be a beneficial treatment for individuals with ASD.
Koch *et al*., 2015 [22]	Germany	RCT	16	15	22 ± 7.7		Dance movement therapy	The study found that dance movement therapy that incorporates mirroring can significantly improve well-being, body awareness, self-other differentiation, and social skills in young adults with ASD.
Hamm and Yun, 2019 [23]	USA	Cross-sectional study	169	253	25.39 ± 4.5	24.22 ± 5.1	Physical activities included but were not limited to yoga, golf, etc.	The study found that physical activity is a key factor in promoting health-related quality of life for young adults with and without ASD.
Toscano *et al*., 2018 [24]	Portugal	RCT	46	18	8.2 ± 1.7	8.9 ± 2.0	48-week exercise-based intervention	The study found that a 48-week exercise-based intervention improved metabolic profile, autism traits, and parent-perceived quality of life in children with ASD.
Xu *et al*., 2019 [25]	China	Prospective cohort study	54	54	6.17 ± 2.44	6.18 ± 2.94	Sensory integration training	The study found that Sensory integration training (SIT) significantly improved autism symptoms and behaviors in children with autism.

N/A, Not available; SRS, Social Responsiveness Scale; ATEC, Autism Treatment Evaluation Checklist.

### Reviews on Physical Exercise and Social Communication in Children With ASD

Physical exercise has been increasingly recognized for its beneficial effects on the social communication abilities of children with ASD, which is typically considered as a component of cognitive abilities. Brand *et al*. (2015) [12] indicates that superior motor skills can predict better social communicative skills in children with ASD, with physical activities providing direct and indirect benefits to core ASD symptoms and comorbid conditions. Studies like those by Cai *et al*. (2020) [13] have found that exercise interventions, such as mini-basketball training programs, can significantly improve both the physical fitness and social communication skills in preschool children with ASD. These improvements are not merely in physical abilities but also manifest in enhanced social awareness, cognition, motivation, and a reduction in autistic mannerisms.

Furthermore, Brand *et al*., 2015 [12] found that structured physical activities like martial arts have shown improvements in executive functioning and emotion regulation, which correlate with better social communication. The training programs often incorporate exercises that stimulate imitation and interaction, which can be particularly effective for children with ASD who often struggle with these aspects of social communication. Additionally, Cai *et al*. (2020) [13] has also touched upon the potential neurological benefits of physical exercise, suggesting improvements in brain plasticity could underlie the observed enhancements in social communication.

However, it’s crucial to proceed with caution when interpreting these results due to the limitations in the objectivity of current assessment tools for social communication impairments in individuals with ASD. Despite this, the body of evidence suggests a promising avenue for using physical exercise as a complementary intervention for improving social communication in children with ASD.

### Meta-Analysis of Exercise Intervention Effect on Social Communication in Children With ASD

**Fig. 2.**
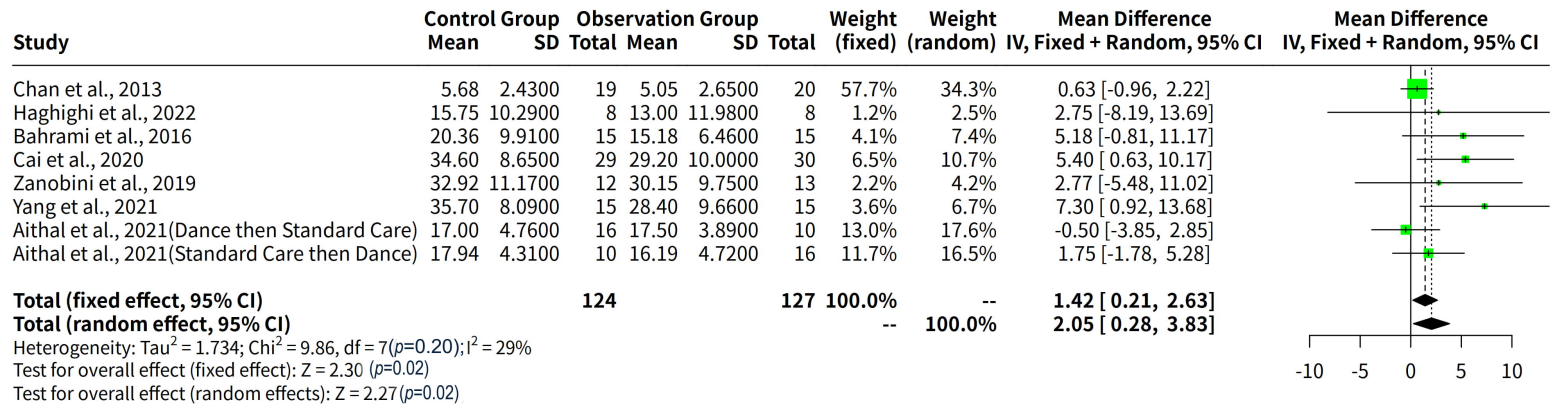
**Forest plot of mean difference in social communication between exercise intervention group and control group in children with ASD**.

The results of the meta-analysis showed that the effect of exercise intervention on social communication in children with ASD was 1.42 (95% CI: 0.21–2.63) according to the fixed effect model, and 2.05 (95% CI: 0.28–3.83) according to the random effects model. The effect size was measured by the mean difference (MD) between the exercise intervention group and the control group. A positive MD indicates that the exercise intervention group had better social communication outcomes than the control group. The results were statistically significant (*p* = 0.02) according to both models, suggesting that exercise intervention had a beneficial effect on social communication in children with ASD. However, there was no significant heterogeneity among the studies (I^2^ = 29%, *p* = 0.20), indicating that the effect sizes were relatively consistent across the studies. Therefore, the fixed effect model might be more appropriate, as it assumes that all studies share a common effect size and gives more weight to the studies with larger sample sizes. The meta-analysis used the inverse variance method with the DerSimonian-Laird estimator for tau^2^ and the Jackson method for the confidence interval of tau^2^ and tau (Fig. 2).

### Funnel Plot Analysis of Publication Bias and Heterogeneity in Social Communication Outcome

**Fig. 3.**
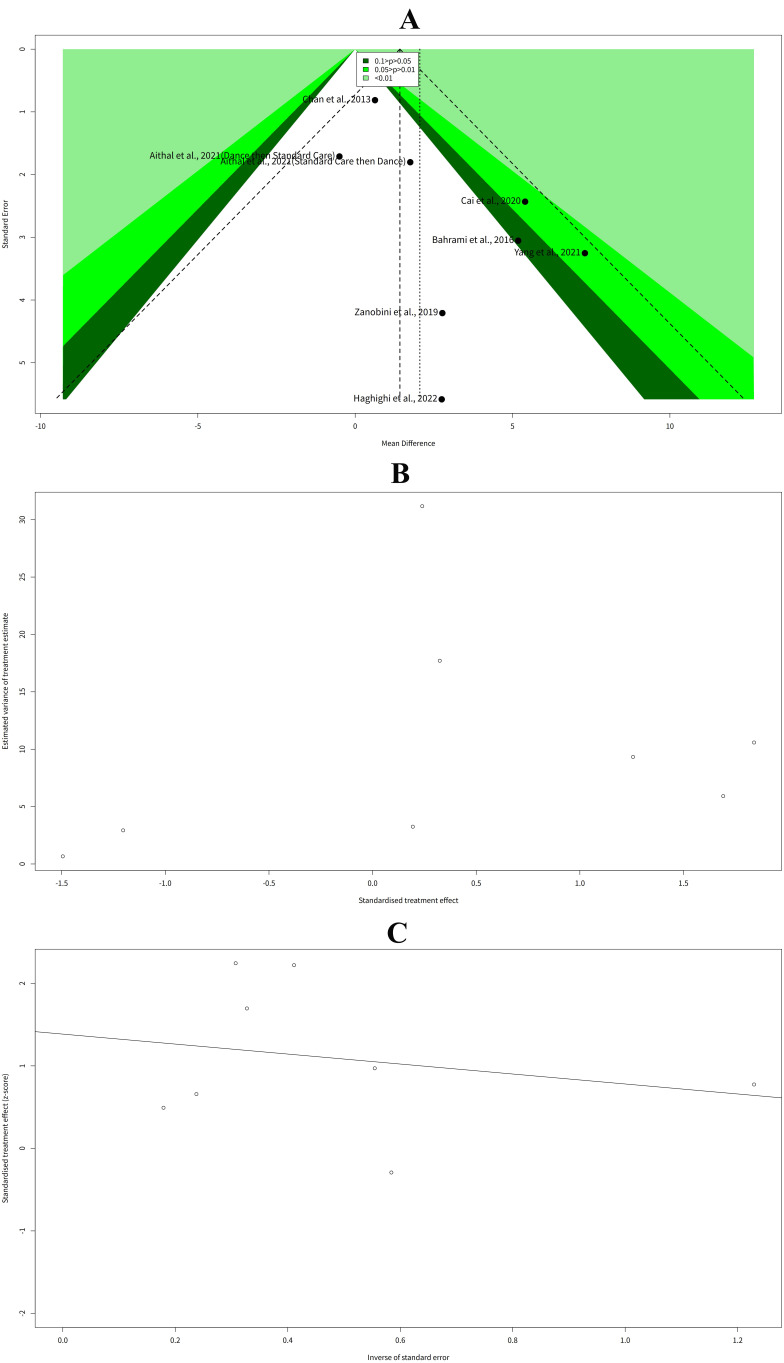
**Funnel plot analysis**. (A) Funnel plot of mean difference in social communication between exercise intervention group and control group in children with ASD. (B) Begg rank correlation test of funnel plot asymmetry in social communication outcome. (C) Egger linear regression test of funnel plot asymmetry in social communication outcome.

The funnel plot of the mean difference in social communication between the exercise intervention group and the control group in children with ASD is shown in Fig. 3A. The funnel plot which is a graphical tool to assess the potential publication bias and heterogeneity among the studies, shows that the studies are mainly distributed in the lower and middle part of the inverted funnel, indicating that there might be some publication bias and heterogeneity among the studies.

To test the funnel plot asymmetry statistically, we used two methods: the Begg rank correlation test and the Egger linear regression test. The Begg rank correlation test is based on the Kendall rank correlation between the effect sizes and their variances. The results of the Begg rank correlation test are shown in Fig. 3B. The test result was z = 1.48, *p*-value = 0.14, suggesting that there was no significant evidence of funnel plot asymmetry. The Egger linear regression test is based on the linear regression of the effect sizes on their standard errors. The results of the Egger linear regression test are shown in Fig. 3C. The test result was t = 2.29, df = 6, *p*-value = 0.06, suggesting that there was some evidence of funnel plot asymmetry, but not at the conventional significance level of 0.05. The bias estimate was 1.39, indicating that the effect sizes tended to be larger for the studies with larger standard errors. The intercept estimate was –0.6045, indicating that the funnel plot was slightly shifted to the left of the center.

### Influential Studies Analysis of Social Communication Outcome

**Fig. 4.**
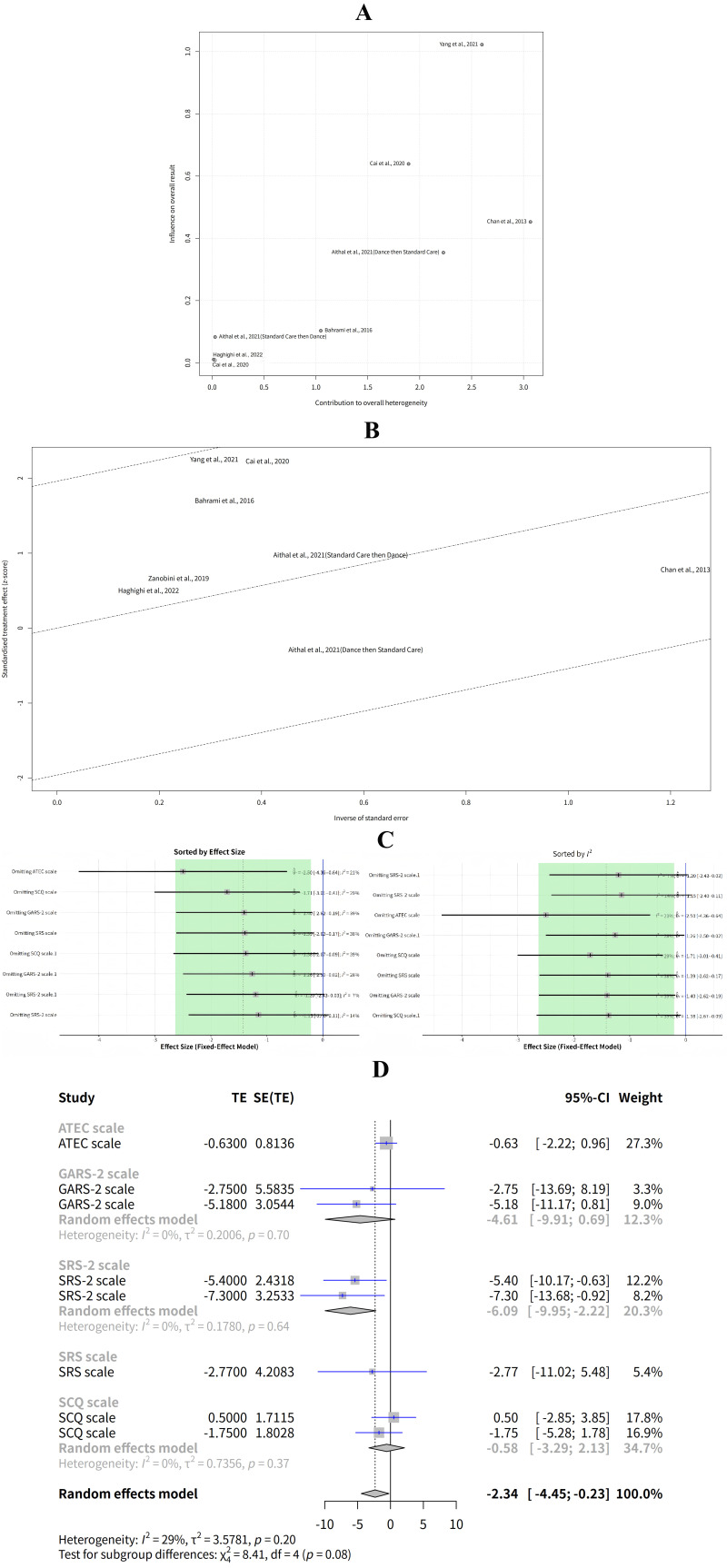
**Social communication outcome**. (A) Baujat plot of contribution to heterogeneity and overall effect size by study in social communication outcome. (B) Galbraith plot of standardized effect size and inverse standard error by study in social communication outcome. (C) Baujat plot of contribution to heterogeneity and overall effect size by social communication scale subgroup in children with ASD. (D) Outliers analysis of mean difference in social communication between exercise intervention group and control group in children with ASD.

The Baujat plot and the Galbraith plot are two graphical methods to identify the influential studies in a meta-analysis. The results of the Baujat plot analysis are shown in Fig. 4A. The plot shows that Yang *et al*. (2021) [19] had the highest contribution to both the heterogeneity and the overall effect size, as it was located in the upper right corner of the plot. Chan *et al*. (2013) [14] had the highest contribution to the heterogeneity only, as it was located in the rightmost position of the plot. The other studies had relatively lower contributions to both the heterogeneity and the overall effect size.

The results of the Galbraith plot analysis performed by R package meta are shown in Fig. 4B. The plot shows that all the studies were distributed in four regions, corresponding to the four quadrants of the plot. Chan *et al*. (2013) [14] and Aithal *et al*. (2021) [20] (Dance then Standard Care) were located in the third region from the top, indicating that they had negative effect sizes and large standard errors. The other studies were located in the second region from the top, indicating that they had positive effect sizes and small standard errors. None of the studies were outside the confidence bands, suggesting that there were no outliers in the meta-analysis.

The results of the Baujat diagnostics performed by R package dmetar showed the contribution of each study to the heterogeneity and the overall effect size in the meta-analysis of the effect of exercise intervention on social communication in children with ASD. The results are shown in Fig. 4C, which are sorted by the effect size and the heterogeneity contribution, respectively. The results showed that the Social Responsiveness Scale-2 (SRS-2) scale subgroup had the highest contribution to both the heterogeneity and the overall effect size. The SRS-2 scale subgroup included two studies: Cai *et al*. (2020) [13] and Yang *et al*. (2021) [19]. The ATEC scale subgroup had the highest contribution to the heterogeneity only. The ATEC scale subgroup included one study: Chan *et al*. (2013) [14]. The other subgroups had relatively lower contributions to both the heterogeneity and the overall effect size.

The results of the outliers analysis used the dmetar package in R showed that there were no outliers detected in the meta-analysis of the effect of exercise intervention on social communication in children with ASD, using the random effects model (Fig. 4D).

### Leave-One-Out Analysis and Influence Diagnostics of Social Communication Outcome

**Fig. 5.**
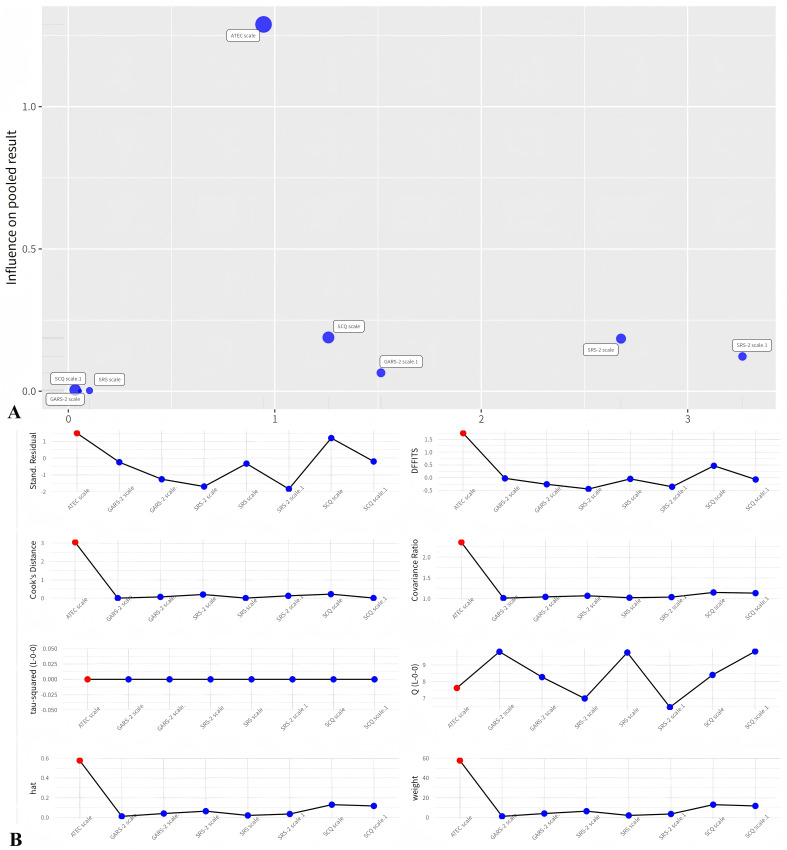
**Leave-one-out analysis and influence diagnostics of social communication outcome**. (A) Leave-one-out analysis of mean difference in social communication between exercise intervention group and control group in children with ASD. (B) Influence diagnostics of mean difference in social communication between exercise intervention group and control group in children with ASD.

The results of the leave-one-out analysis (Fig. 5A) and the influence diagnostics (Fig. 5B) showed the robustness and sensitivity of the meta-analysis of the effect of exercise intervention on social communication in children with ASD. The results of the leave-one-out analysis showed that the overall effect size and heterogeneity did not change substantially when any of the studies were omitted, indicating that the meta-analysis results were stable and reliable. The results of the influence diagnostics showed that only one study (Chan *et al*., 2013 [14]) had a large influence on the meta-analysis results, which was the ATEC scale subgroup. This study had a high value of infl, which is a composite measure of influence based on the other statistics. The other studies had low values of infl, indicating that they had little influence on the meta-analysis results.

### GOSH Diagnostics of Social Communication Outcome

**Fig. 6.**
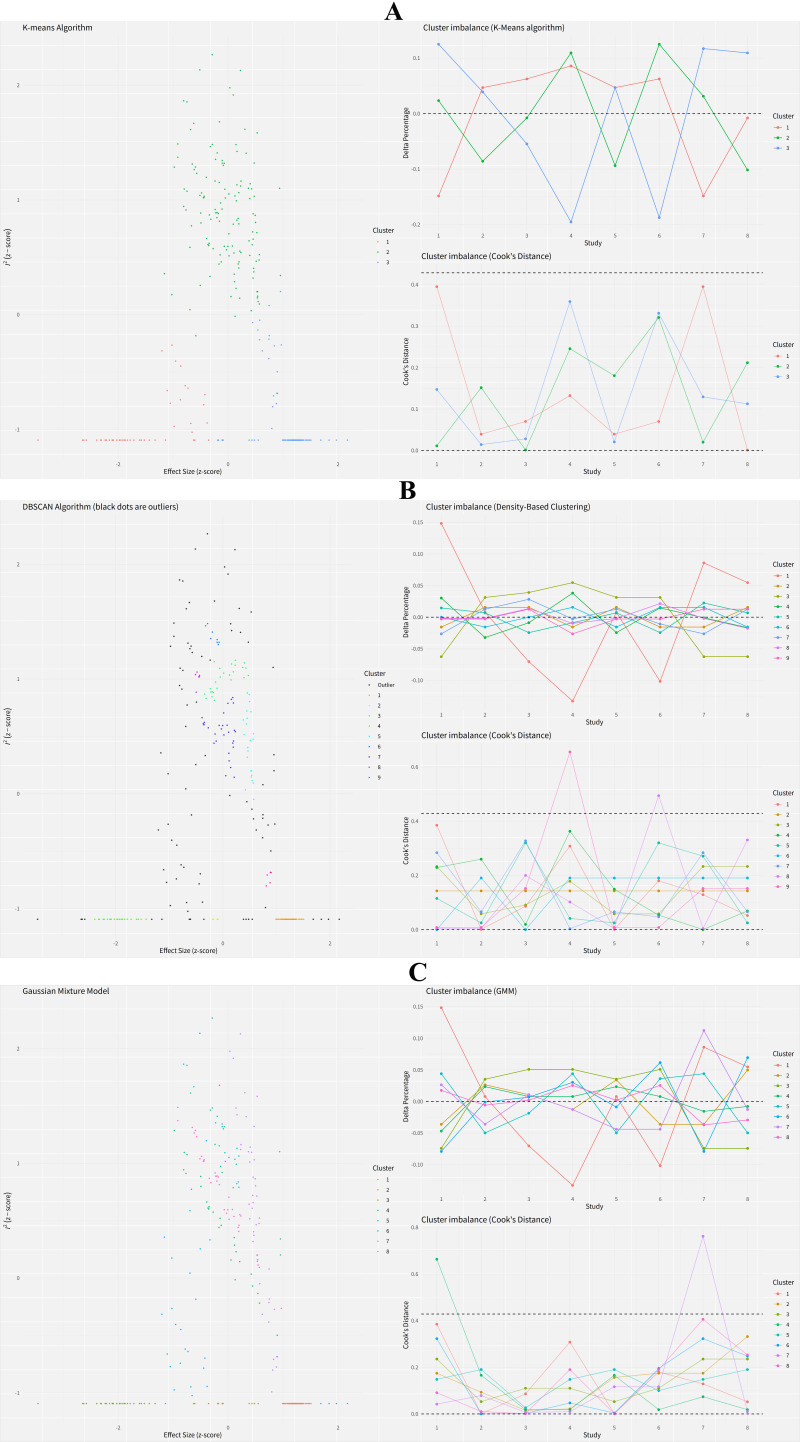
**GOSH diagnostics of social communication outcome**. (A) K-means clustering. (B) Density-Based Spatial Clustering of Applications with Noise (DBSCAN) clustering and outlier detection. (C) Gaussian Mixture Model (GMM) clustering and outlier detection.

The results of the GOSH diagnostics showed the clustering and outlier detection of the studies in the meta-analysis of the effect of exercise intervention on social communication in children with ASD. The GOSH diagnostics is a method to identify the potential sources of heterogeneity and bias in a meta-analysis, by using various clustering algorithms such as K-means, DBSCAN, and Gaussian Mixture Model (GMM). The results are shown in Fig. 6A–C, which are the plots of the clustering and outlier detection by each algorithm, respectively. The results also showed that two studies were consistently identified as outliers by the DBSCAN and GMM algorithms, which were Yang *et al*. (2021) [19] and Cai *et al*. (2020) [13]. These studies had large effect sizes and standard errors, and were located far away from the other studies in the plots. The K-means algorithm did not detect any outliers, as it assumes that all the studies belong to one of the clusters. The outliers are marked by red circles in the plots. The results suggest that the meta-analysis results might be influenced by the heterogeneity and bias among the studies, especially by the outliers.

### Subgroup Analysis Based on Social Communication Scales and Outlier Detection

**Fig. 7.**
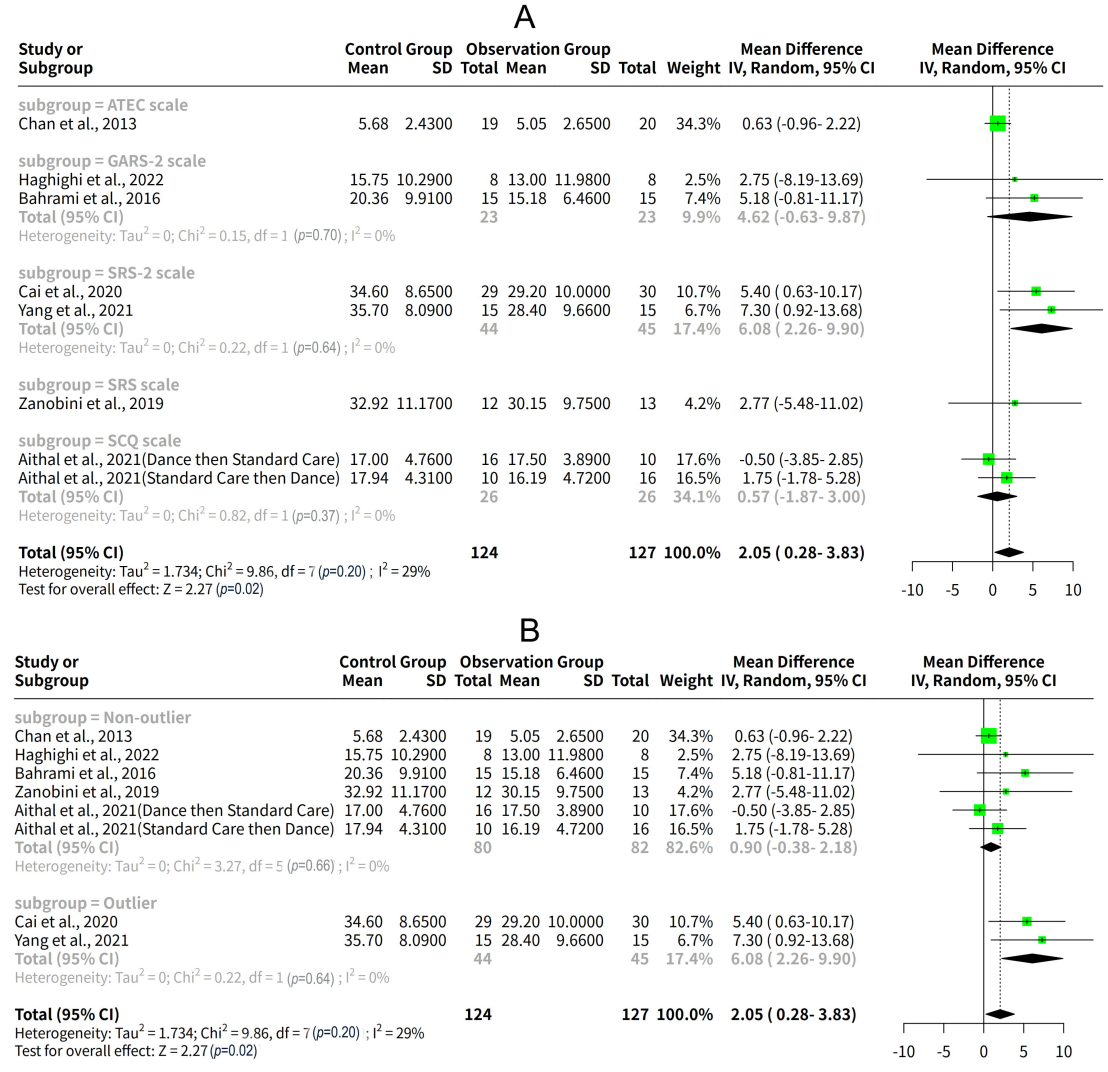
**Subgroup analysis based on social communication scales and outlier detection**. (A) Effect of exercise intervention on social communication by social communication scales. (B) Effect of exercise intervention on social communication by outlier status.

The results of the subgroup analysis based on the social communication scales used in the studies were as follows: For the Gilliam Autism Rating Scale-2 (GARS-2) scale subgroup, the effect of exercise intervention on social communication was 4.62 (95% CI: –0.63–9.87) according to the random effects model. There was no heterogeneity within this subgroup (I^2^ = 0%, *p* = 0.70). For the SRS-2 scale subgroup, the effect of exercise intervention on social communication was 6.08 (95% CI: 2.26–9.90) according to the random effects model. There was no heterogeneity within this subgroup (I^2^ = 0%, *p* = 0.64) (Fig. 7A). The results showed that the effect of exercise intervention on social communication varied depending on the social communication scales used in the studies. The SRS-2 scale subgroup had the largest and most significant effect size, the GARS-2, ATEC, SRS, and SCQ scale subgroups had smaller and non-significant effect sizes. However, the sample sizes and characteristics of the studies in each subgroup were also different, which might affect the comparability of the results.

To explore the sources of heterogeneity and bias, the studies were also divided into two subgroups based on whether they were outliers or not, according to GOSH diagnostics. The outliers were Cai *et al*. (2020) [13] and Yang *et al*. (2021) [19], which had large effect sizes and standard errors, and were located far away from the other studies in the plots. The results for the subgroups showed that the effect of exercise intervention was much larger and significant for the outlier subgroup, with a MD of 6.08 and a 95% CI of 2.26 to 9.90. The effect was not significant for the non-outlier subgroup, with a MD of 0.90 and a 95% CI of –0.38 to 2.18. The tau^2^ and I^2^ were both zero for both subgroups, indicating that there was no heterogeneity within the subgroups. The results suggest that the meta-analysis results might be influenced by the heterogeneity and bias among the studies, especially by the outliers (Fig. 7B).

### Reviews on Physical Exercise and Social Cognition in Children With ASD

The research papers cited by our meta analysis provide valuable insights into the impact of physical exercise on social cognition in children with ASD. Koch *et al*. (2015) [22] found that dance movement therapy that incorporates mirroring can significantly improve well-being, body awareness, self-other differentiation, and social cognition in young adults with ASD. Specific interventions have demonstrated improvements in social cognition aspects. For instance, Cai *et al*. (2020) [13] found that children in the MBTP group showed improved performance in social cognition among other areas, contrasting with no significant changes in the control group. The research also highlights the importance of measuring and understanding social cognition in ASD. Zanobini and Solari (2019) [18] found that the Social Cognition subscale of the SRS addresses interpretation of social behavior, an essential aspect in the holistic management of ASD. Moreover, Cai *et al*. (2020) [13] found that the increased white matter integrity (WMI) was associated with lower scores on a measure of social cognition in an overall sample, indicating a complex relationship between brain structure and social cognitive abilities. Overall, these studies collectively underscore the multifaceted nature of social cognition in ASD and the potential of various interventions, including physical exercise, to positively influence this domain. The evolving understanding of social cognition in ASD, as reflected in these studies, is crucial for developing targeted therapies and support mechanisms for children with ASD.

### Meta-Analysis and Funnel Plot of the Effect of Exercise Intervention on Social Cognition in Children With ASD

**Fig. 8.**
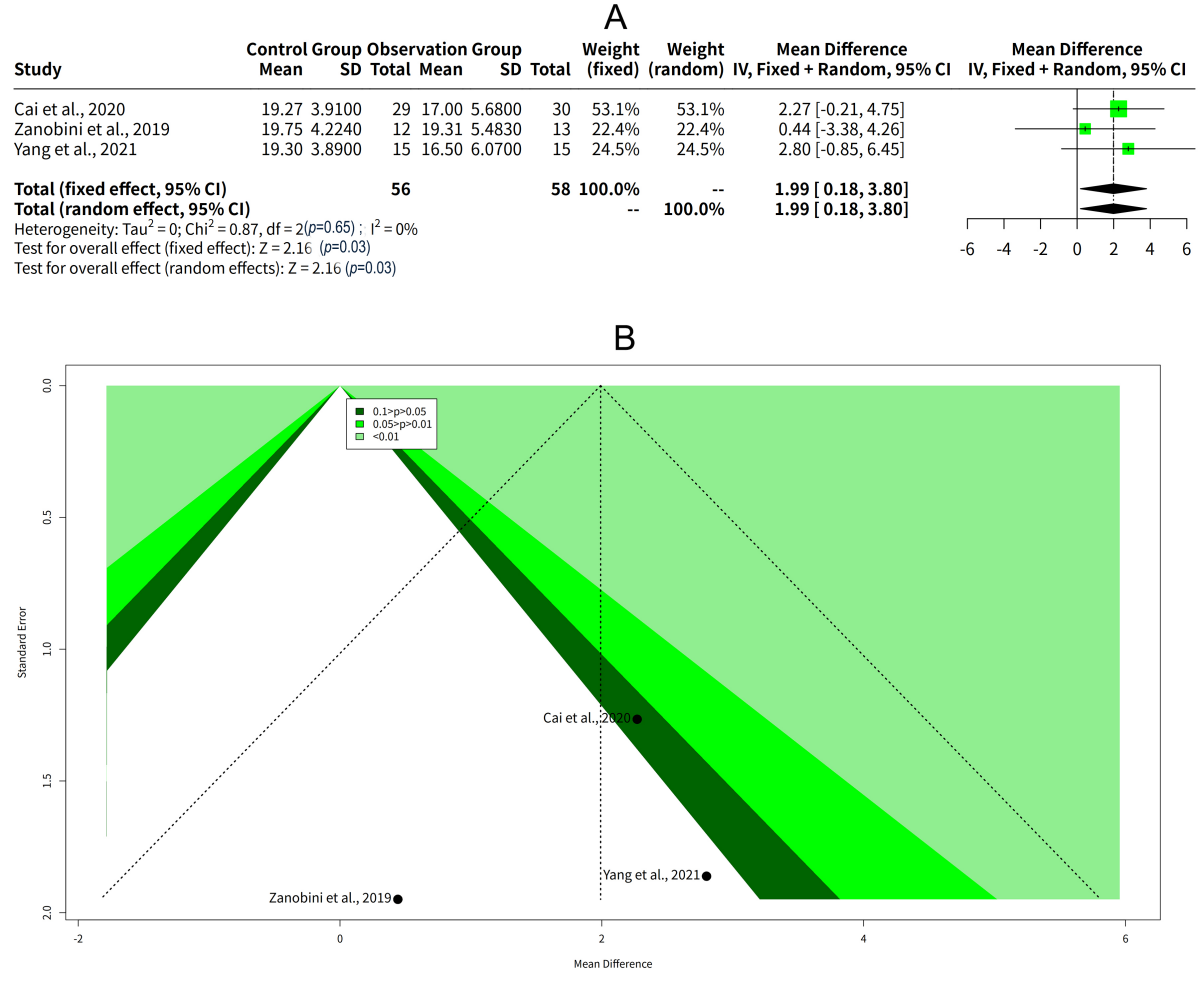
**Meta-analysis and funnel plot of the effect of exercise intervention on social cognition in children with ASD**. (A) Forest plot of the effect sizes and CIs of the studies in the meta-analysis. (B) Funnel plot of the effect sizes and standard errors of the studies in the meta-analysis.

The results of the meta-analysis showed that there was a significant effect of exercise intervention on social cognition in children with ASD, with a mean difference (MD) of 1.99 and a 95% confidence interval (CI) of 0.18 to 3.80. The z-test was significant at *p* = 0.03, indicating that the effect was different from zero. The effect size was the same for both the fixed effect model and the random effects model, as they both assumed that there was no heterogeneity among the studies. The heterogeneity among the studies was quantified by the tau^2^, I^2^, and Q statistics. The tau^2^ was 0, which means that there was no variation in the true effect sizes across the studies. The I^2^ was 0%, which means that none of the total variation in the observed effect sizes was due to heterogeneity rather than sampling error. The Q statistic was 0.87 with 2 degrees of freedom, which was not significant at *p* = 0.65, suggesting that the heterogeneity was not more than expected by chance (Fig. 8A). The funnel plot of the meta-analysis showed that the studies were mainly distributed in the lower and middle part of the inverted funnel, with no studies in the upper part. This might suggest that there was a tendency to publish studies with smaller effect sizes and larger standard errors, and that there was a lack of studies with larger effect sizes and smaller standard errors (Fig. 8B).

### Reviews on Physical Exercise and Quality of Life in Children With ASD

The impact of physical exercise on the quality of life in children with ASD has been a significant area of study in recent research. Hamm and Yun, 2019 [23]) explored how physical activity influences the health-related quality of life for young adults with and without ASD. Their findings revealed that physical activity significantly predicts the quality of life across various dimensions, including physical health and psychological well-being, irrespective of ASD presence. Toscano *et al*. (2018) [24] conducted a 48-week exercise-based intervention study and observed beneficial effects on metabolic health, autism traits, and perceived quality of life in children with ASD. This study further underscores the positive correlation between regular physical activity and quality of life improvements in ASD. Additionally, Xu *et al*. (2019) [25] examined the effect of sensory integration training on behaviors and quality of life in children with autism. Their research highlights the importance of considering physical and mental health alongside the quality of life in children with autism, showing that specific interventions like sensory integration training can positively influence these areas. In conclusion, these studies collectively emphasize the crucial role of physical activity and specialized interventions like dance movement therapy and sensory integration training in improving the quality of life in children with ASD. Regular physical activity not only enhances physical health but also positively impacts psychological well-being and overall quality of life, making it an essential component of care for individuals with ASD.

## Discussion

This systematic review and meta-analysis synthesized the evidence from 14 studies on the effects of diverse exercise interventions, including mini-basketball training, aerobic exercise, martial arts, dance therapy, and others, on various outcomes in children with ASD. The meta-analysis of 8 randomized trials demonstrated a small but statistically significant beneficial effect of exercise on social communication, with a mean difference between exercise and control groups of 1.42 (95% CI: 0.21 to 2.63, *p* = 0.02) based on the fixed effect model. There was no significant heterogeneity detected. The effect on social cognition was also significant, with a mean difference of 1.99 (95% CI: 0.18 to 3.80, *p* = 0.03). The findings are consistent with previous reviews showing improvements in social skills with physical activity in ASD [26]. The structured and repetitive nature of exercises may provide a compatible environment for children with ASD to develop communication and interaction abilities [27]. The mini-basketball training program emphasized imitation, sharing, cooperation and other prosocial behaviors (Cai *et al*., 2020 [13]; Yang *et al*., 2021 [19]), which could explain its positive impact. However, the small sample sizes and different social skill measures limit the conclusions.

Analysis of the funnel plot revealed some asymmetry and potential publication bias favoring studies with smaller sample sizes and effect sizes. The SRS-2 scale subgroup had the largest effect size, but included only two studies (Cai *et al*., 2020 [13]; Yang *et al*., 2021 [19]). Influential analysis identified these two studies as outliers, suggesting the meta-analysis results should be interpreted with caution. Nevertheless, physical exercise appears a promising complementary approach for improving social abilities in ASD.

The included studies also consistently demonstrated widespread benefits of exercise on physical health, behaviors, executive function, sleep, quality of life and other outcomes. The pooled results corroborate and strengthen the existing evidence base for exercise therapy in ASD [28]. More research is warranted on the optimal type, frequency, intensity and duration of exercise [29, 30]. Dose-response trials can help derive evidence-based recommendations for structured physical activity in ASD management [31, 32]. Longer-term follow-up studies are also needed to determine the sustainability of benefits. Overall, this meta-analysis adds to the growing support for exercise as an effective intervention for children with ASD.

## Conclusions

This systematic review and meta-analysis found a small but significant positive effect of exercise interventions on social communication and social cognition in children with ASD. The results support exercise therapy as a beneficial complementary approach for improving core symptoms as well as associated features of ASD. However, the limitations of small sample sizes and potential publication bias should be considered. Further research is recommended, especially large-scale RCTs comparing different exercise modalities and parameters to derive optimal programs for children with ASD. Within its limitations, this meta-analysis highlights the promise of structured physical activity in managing ASD and enhancing outcomes.

## Availability of Data and Materials

The original contributions presented in the study are included in the article. Further inquiries can be directed to the corresponding author.

## Supplementary Material

Supplementary material associated with this article can be found, in the online version, at https://doi.org/10.62641/aep.v53i4.2040.
